# Virtual Alanine Scan of the Main Protease Active Site in Severe Acute Respiratory Syndrome Coronavirus 2

**DOI:** 10.3390/ijms22189837

**Published:** 2021-09-11

**Authors:** Tomoki Nakayoshi, Koichi Kato, Eiji Kurimoto, Akifumi Oda

**Affiliations:** 1 Graduate School of Information Sciences, Hiroshima City University, 3-4-1 Ozukahigashi, Asaminami-ku, Hiroshima 731-3194, Hiroshima, Japan; nakayoshi@hiroshima-cu.ac.jp; 2 Faculty of Pharmacy, Meijo University, 150 Yagotoyama, Tempaku-ku, Nagoya 468-8503, Aichi, Japan; kato-k@kinjo-u.ac.jp (K.K.); kurimoto@meijo-u.ac.jp (E.K.); 3 Faculty of Pharmaceutical Sciences, Shonan University of Medical Sciences, 16-48 Kamishinano, Totsuka-ku, Yokohama 244-0806, Kanagawa, Japan; 4 Institute for Protein Research, Osaka University, 3-2 Yamadaoka, Suita 565-0871, Osaka, Japan

**Keywords:** COVID-19, severe acute respiratory syndrome coronavirus 2, main protease, drug resistance, virtual alanine scan, molecular dynamics simulation

## Abstract

Recently, inhibitors of the severe acute respiratory syndrome coronavirus 2 (SARS-CoV-2) main protease (Mpro) have been proposed as potential therapeutic agents for COVID-19. Studying effects of amino acid mutations in the conformation of drug targets is necessary for anticipating drug resistance. In this study, with the structure of the SARS-CoV-2 Mpro complexed with a non-covalent inhibitor, we performed molecular dynamics (MD) simulations to determine the conformation of the complex when single amino acid residue in the active site is mutated. As a model of amino acid mutation, we constructed mutant proteins with one residue in the active site mutated to alanine. This method is called virtual alanine scan. The results of the MD simulations showed that the conformation and configuration of the ligand was changed for mutants H163A and E166A, although the structure of the whole protein and of the catalytic dyad did not change significantly, suggesting that mutations in His163 and Glu166 may be linked to drug resistance.

## 1. Introduction

Severe acute respiratory syndrome coronavirus 2 (SARS-CoV-2) is the causative agent of COVID-19. SARS-CoV-2 is a positive-sense single-stranded RNA virus, with its genome decoded by Wu et al. [1]. The SARS-CoV-2 genome contains 11 open reading frames, one of which encodes the nonstructural protein ORF1ab. The main protease (Mpro) is a protease that is also a part of ORF1ab and cleaves ORF1ab. The structure of the SARS-CoV-2 protein has been studied intensely from 2020 to 2021, and the structure of SARS-CoV-2 Mpro was solved in February 2020.

As a means of combating the COVID-19 pandemic, development of therapeutic agents is being actively pursued alongside vaccine development [2,3]. For example, the SARS-CoV-2 RNA-dependent RNA polymerase [4], spike protein [5], 2′-*O*-ribose methyltransferase [6], and human angiotensin-converting enzyme 2 [7,8] have been investigated as candidate anti-SARS-CoV-2 drug targets, as well as SARS-CoV-2 Mpro [9,10]. SARS-CoV-2 Mpro is an essential enzyme in the production of viral proteins and cleaves peptide chains after glutamine residues; no human protease has been found to exhibit similar substrate specificity. Therefore, Mpro is a promising drug target for anti-SARS-CoV-2 [9]. Because proteases are drug targets of other antiviral drugs, such as anti-HIV drugs, there have been attempts to use these drugs as anti-SARS-CoV-2 drugs by drug repositioning [11]. However, unlike many other viral proteases, SARS-CoV-2 Mpro is a cysteine protease, and drug repositioning has not been successful.

SARS-CoV-2 Mpro has high homology with other coronavirus (e.g., SARS-CoV and MERS-CoV) main proteases [9,12] and is structurally similar. SARS-CoV-2 Mpro contains His41 and Cys145 as catalytic dyads in the center of the active site. In the proteolytic process of Mpro, the proton from the Cys145 side chain is first withdrawn by His41, and the resulting thiolate exerts a nucleophilic attack on the peptide bond of the substrate. The N-terminal side peptide withdraws a proton from histidine and is released from the enzyme-product complex. The hydrolysis of the thiolate then releases the C-terminal side peptide. Mpro is the homodimer and functions mainly as an enzyme. Although some Mpro mutants form monomers or “immature” dimers without the interaction at the homodimer interface, their enzymatic activities are lower than Mpro homodimer’s one [13]. For example, E166A mutant lacks the interaction between E166 and S1 at the homodimer interface, but shows enzymatic activity [13]. In addition, in the crystal structure (PDB ID: 2duc), SARS-CoV Mpro is enzymatically active despite the lack of hydrogen bonds between E166 in B chain and S1 in A chain [14]. In addition to the active site, the homodimer interface is also considered a possible binding site for SARS-CoV-2 Mpro inhibitors, but, at present, inhibitors for the active site are predominantly being studied.

So far, covalent and non-covalent inhibitors have been identified as possible SARS-CoV-2 Mpro inhibitors. The covalent inhibitors PF-07304814 and PF-07321332 [15] are already in clinical trials as oral and injectable drugs, respectively, and are expected to be candidates in the treatment of COVID-19. PF-00835231 [16] and GC-376 [17], expected to be anti-SARS-CoV-2 agents, are also covalent inhibitors that covalently bind to the Cys145 side chain of the catalytic dyad in the active center of Mpro, thereby inhibiting the function of Mpro. PF-00835231 in particular has been suggested to function as a pan-coronavirus inhibitor in inhibition assays against various viral proteases, while viral inhibition assays of PF-00835231 have shown that antiviral activities were not observed for two human rhinovirus strains (HRV-14 and HRV-16), human immunodeficiency virus-1, human cytomegalovirus, and HCV replicon. Therefore, all of these covalent inhibitors are expected to be selective anti-SARS-CoV-2 drugs. Non-covalent inhibitors are also being investigated, and Douangamath et al. found 48 covalent inhibitors and 23 non-covalent inhibitors by fragment screening [18]. Of these, the crystal structure of NCL-00024905 (PDB ID: 5rg1) has been subject to algebraic topology and deep learning methods in order to predict binding affinity and analyze interactions. In 5rg1, NCL-00024905 is bound to the active site in Mpro. Results of algebraic topology and deep learning have suggested that the binding affinity of NCL-00024905, a non-covalent inhibitor, is comparable to that of covalent inhibitors. These results are significant because the discovery of non-covalent inhibitors alongside covalent inhibitors is beneficial for avoiding toxicity.

In this study, we performed molecular dynamics (MD) simulations of the complex structure of SARS-CoV-2 Mpro with the non-covalent inhibitor NCL-00024905. For Mpro, artificial mutants were constructed by replacing one amino acid residue in the active site with alanine (Ala). Then, MD simulations were performed to determine which residues of Mpro played important roles in the recognition of NCL-00024905 and which mutations prevented inhibitor recognition. We also identified residues that do not disrupt the structure of the catalytic dyad, even after substitution. The amino acid mutation that both prevent inhibitor recognition and maintain the structure of catalytic dyad may confer viral drug resistance, making our study useful in predicting drug-resistant mutants.

To date, a number of MD simulations of SARS-CoV-2 Mpro have been performed. MD simulations of Mpro in complex with substrate-mimic peptides [19] have shown that the catalytic dyad of monomeric Mpro was not suitable for enzymatic reactions, suggesting the importance of dimer formation in Mpro function. Although unrelated to drug resistance, MD simulations of naturally occurring mutants of Mpro have also been performed [20], and it has been shown that amino acid mutations cause changes in the protein structure. In addition, we have used MD simulations to predict the 3D structures of mutant proteins, and shown that simulation results can explain experimentally measured enzymatic activities of mutants [21,22,23,24]. Thus, the MD simulations of artificial Mpro mutants are helpful in clarifying structural characteristics of Mpro, and we expect that this study can be used to predict the possible drug resistance.

## 2. Results and Discussion

**Figure 1:**
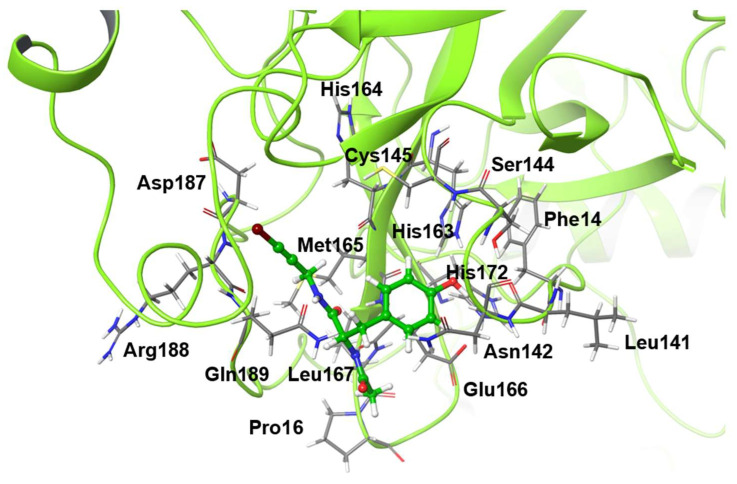
Alanine scanned residues. The molecule drawn as a ball-and-stick model is the inhibitor.

The mutants considered in this study are shown in Table 1, and the residues that were mutated from the wild-type structure are shown in Figure 1. For the virtual alanine scan, all residues within a radius of 4 angstrom (Å) from the ligand in the wild-type structure after 1000-ns MD simulation were contained.

**Figure 2:**
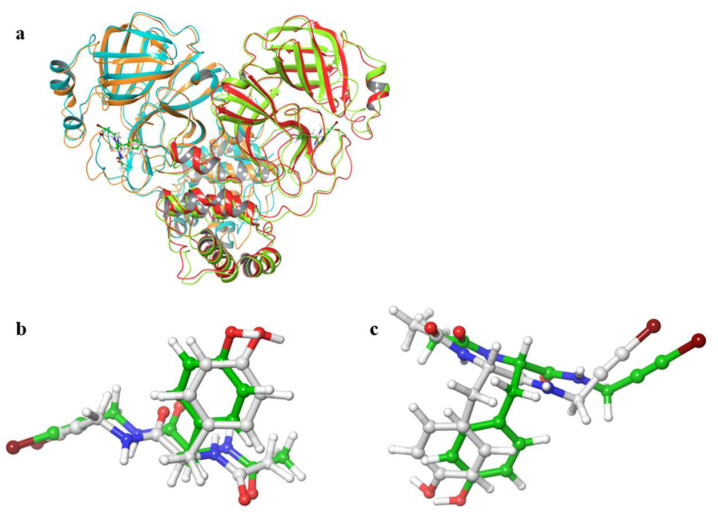
3D structures of SARS-CoV-2 Mpro before and after MD simulations. (**a**) Protein-ligand complex. Red and orange ribbons indicate chains A and B before MD simulations (0 ns), and light green and light blue indicate chains A and B after MD simulations (1000 ns). (**b**) Ligand in chain A. (**c**) Ligand in chain B. White and green ligands are the structures before and after MD simulation, respectively.

**Figure 3:**
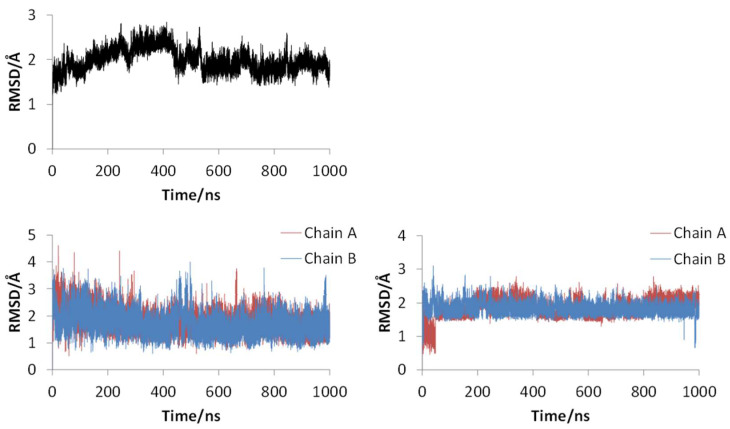
RMSDs for main chain (upper), ligand (lower left), and catalytic dyad (lower right) of wild-type SARS-CoV-2 Mpro.

Figure 2 shows the 3D structures of wild-type SARS-CoV-2 Mpro before and after MD simulation. In addition, Figure 3 shows the main-chain RMSDs, ligand RMSDs, and catalytic dyad RMSDs throughout the wild-type MD trajectory. As shown in these figures, the conformation of the wild-type SARS-CoV-2 Mpro did not change significantly before and after the simulation, and all RMSDs converged. Throughout the simulations, not only the overall structure shown in Figure 2a but also the ligands retained similar structures as shown in Figure 2b, indicating that the 1000-ns MD simulation obtained the equilibrium structure for wild-type Mpro. In particular, the convergence of the catalytic dyad and the ligand RMSDs suggests that Mpro is responsible for the enzymatic reaction and that the experimental structure is reasonable for the pose of the inhibitor. In this study, the calculations were performed in the homodimeric state, and our results are consistent with a previous study suggesting that the catalytic dyad does not change significantly in the homodimeric state [19].

Figures S1 and S2 in the Supplemental Material show the ligand RMSDs and catalytic dyad RMSDs throughout the MD simulation for each mutant. Table 2 summarizes the averaged values of main-chain, ligand, and catalytic-dyad RMSDs in the final 10 ns of trajectories. As shown Figures S1 and S2, main-chain RMSDs were not very large for all mutants, indicating that the structure is not broken, at least throughout the 100-ns MD simulation. However, for some mutants, such as S144A, RMSDs increased throughout the simulation, suggesting a trend towards a conformational change. Indeed, S144A was one of two mutants with RMSDs of the main chain exceeding 1.5 Å. For example, in our previous study, simulations of canine CES2, which is experimentally different from human CES2, did not show complete collapse of the structure. [25] Therefore, evaluating the complete disruption of the structure by MD simulation remains difficult. In this study, we have evaluated the effect of mutation on enzyme function by using catalytic dyad RMSDs together with main-chain RMSDs. Among the mutants studied, the 3D structure of C145A has been experimentally determined. [26] Although C145 is a residue that constitutes the catalytic dyad and plays an important role in enzyme activity, the experimental 3D structure of C145A is not significantly different from that of the wild type. In addition, experimental results have shown that substrate recognition for the mutant is similar to that of the wild type. Our current results show that the RMSDs of the main chain, ligand, and catalytic dyad were all small in C145A, consistent with the experimental results that the mutation of C145A has little effect on the protein structure.

Even in systems with ligand RMSDs greater than 1.5 Å, only one of two ligands had a larger RMSD. In other words, the structure of only one ligand changed significantly from the initial. In N142A and S144A, the ligand bound to the A chain moved significantly, while in H163A and E166A, the ligand bound to the B chain moved significantly. Although SARS-CoV-2 Mpro is a homodimer with high structural symmetry as described above, partial conformational changes are observed in the 100-ns MD simulation. Virtual alanine scans are a simple way to evaluate protein-ligand interactions in the early stages of drug discovery, and it is impractical to run MD simulations long enough to evaluate the symmetry of the entire protein structure. Our results suggest that 100-ns MD simulations can be used to evaluate the effect of mutation by virtual alanine scanning as a first step in drug discovery. In mutants with large ligand RMSDs, the structure of the inhibitor deviates significantly from the initial, wild-type structure, suggesting that the protein-inhibitor interaction of these mutants is weakened compared to the wild type, and that the mutation in the protein prevents the inhibitor from staying in Mpro. Therefore, Mpro may be resistant to NCL-00024905 in these mutants. As shown in Figure S1, the structures of N142A, H163A, and E166A not only deviated from the initial structure in the final frame, but also fluctuated greatly in the middle of the simulation. This data indicates that the protein is not able to bind the ligand strongly in these mutants, perhaps because recognition of the inhibitor is weakened. On the other hand, S144A and M165A showed relatively large values of the main-chain RMSDs, as well as the ligand RMSDs, suggesting that both the ligand structure and structure of the entire system has changed. Thus, not only inhibitor recognition, but also enzymatic function, may be affected by the mutants. In particular, main-chain RMSDs in S144A did not converge, and the structure may be further disrupted in longer simulations. Drug resistance only becomes a problem when a protein retains its function but can no longer be recognized an inhibitor. Mutations that affect the entire protein structure may not be important in the context of drug resistance because the enzyme will no longer function.

**Figure 4:**
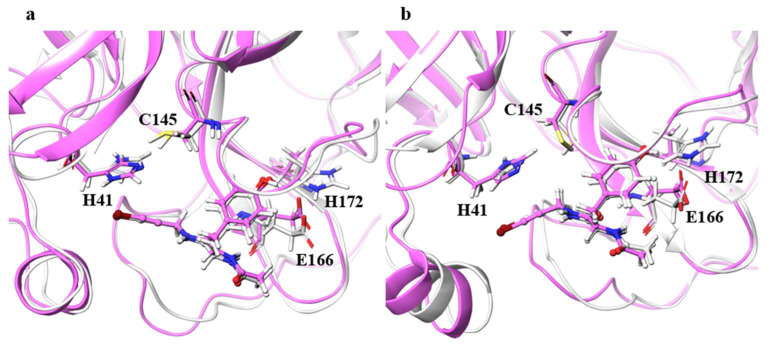
Structures around the ligand in the (**a**) A and (**b**) B chains. Atoms and chains of wild type and H172A are presented in white and magenta, respectively. The ligand is represented by the ball-and-stick model, and H41, C145, E166, and H172 are represented by the stick model.

**Figure 5:**
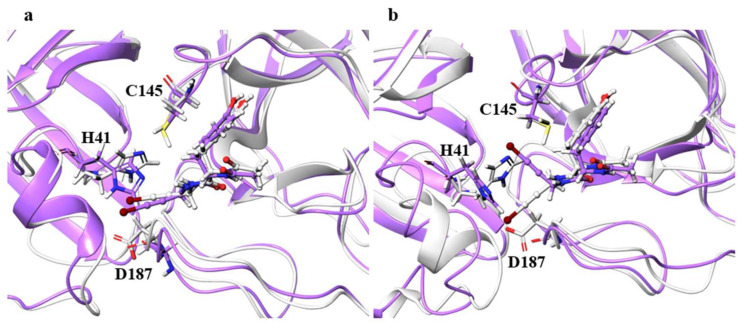
Structures around the ligand in the (**a**) A and (**b**) B chains. Atoms and chains of wild type and D187A are presented in white and purple, respectively. The ligand is represented by the ball-and-stick model, while H41, C145, and D187 are represented by the stick model.

For the catalytic dyad RMSDs, as with the ligand RMSDs, there was a case (H172A) where the catalytic dyad of only one chain underwent a significant conformational change, as shown in Figure 4. It is impractical to simulate the conformational change of both catalytic dyads for a long time, and the 100-ns simulation shows that the conformational change of at least one catalytic dyad can be followed. The catalytic dyad is part of the protein and is covalently linked to other parts of the protein. Thus, the structure of the catalytic dyad tends to be more constrained than that of the non-covalent ligand. However, the structures of H172A and D187A differed from the wild type. In particular, the structure of the catalytic dyad in D187A changed in both the A and B chains as shown in Figure 5, and the complex structure did not converge in the 100-ns simulation as shown in Figure S2. In addition, the structure of the ligand docked with the B chain also changed in D187A. These results indicate that the structure of entire active site, including both catalytic dyad and ligand, changed in D187A. If the structure of the entire active site is changed, SARS-CoV-2 Mpro may lose its function, in which case SARS-CoV-2 would not be able to proliferate. Because H172A and D187A are mutations that weaken the function of the enzyme itself and affect the recognition of inhibitors, they may not be as important for drug resistance, same as S144A and M165A mentioned above. H172 is an amino acid facing the interchain interface and is in position to form an electrostatic interaction with E166. Because E166 forms a hydrogen bond with NCL-00024905, H172 is involved in both dimer formation and ligand recognition, perhaps causing the structure of the catalytic dyad to change significantly in H172A. D187, on the other hand, is an exposed residue near the protein surface and is the acidic amino acid closest to H41 that forms the catalytic dyad. Since there are charged amino acids including R40 and H164 around H41 and D187, the loss of charge at this site significantly affected the electrostatic environment of the catalytic dyad and its structure. Although the final RMSD of H164A was small, the catalytic dyad of the B chain fluctuated to a large degree in the middle of the simulation, indicating a temporary change in the dynamic structure. Although the mutant H164A did not show significant changes in ligand recognition and the final RMSD, as well as the dynamic structure, (RMSF) did not differ much from the wild type, and the fluctuation of the catalytic dyad may affect enzymatic activity.

**Figure 6:**
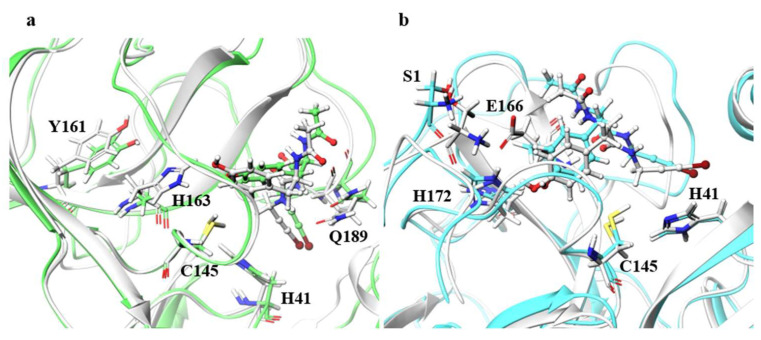
Structures around the ligand in the B chain. (**a**) Superimposition of the wild type and H163A around the ligand. Only the B chain is illustrated. Atoms and chains of wild type and H163A are presented in white and light green, respectively. The ligand is represented as a ball-and-stick model, and H41, C145, Y161, H163, and Q189 are represented by the stick model. (**b**) Superimposition of the wild type and E166A. Both A and B chains are illustrated. Atoms and chains of wild type and E166A are presented in white and cyan, respectively. Ligands are represented by the ball-and-stick models, and S1, H41, Cys145, E166, and H172 are represented by stick model. Of these residues, Only S1 belongs to the A chain, and the other residues belong to the B chain.

Although the ligand RMSDs of N142A, H163A, E166A, and R188A were large, the structures of the main chain and the catalytic dyad were not significantly affected. Especially for H163A and E166A, final RMSDs of the ligands shown in Table 2 exceeded 2.0 Å, and the ligand RMSD values fluctuated during MD trajectories as shown in Figure S1. Figure 6 illustrates the residues around the ligand in the B chain of H163A and in the B chain of E166A. For H163A, the structure of the catalytic dyad was almost unchanged from the wild type, and the protein structure around the catalytic dyad was similar to that of the wild type. Although the catalytic dyad is in a loop structure and there is a residue with a high RMSF nearby, the catalytic dyad structure is conserved with the wild type in H163A. On the other hand, the structure near the protein surface differed from that of the wild type, and the structure of the loop constituting the pocket near the surface was different than the wild type. This structural change was caused by entry of the Y161 side chain into the space vacated by the replacement of H163 with Ala. The migration of the Y161 side chain caused the β-sheet structure to shift, deforming the loop. The change in the pocket structure caused a change in conformation and configuration of the ligand, resulting in a large ligand RMSD. In fact, the RMSF of the ligand was larger than those of wild type by more than 0.1 Å, indicating that the ligand fluctuates in the pocket of the H163A mutant. Similar changes were also observed in the H163A A chain, in which the ligand RMSD was not as large, and a decrease in hydrogen-bonding frequency was observed in the A chain. Both the B chain, in which conformational change of the ligand was observed, and the A chain, in which hydrogen bond cleavage was observed, showed a tendency for the ligand to detach from the protein, indicating that the mutation H163A affected inhibitor recognition. The structure of the catalytic dyad in E166A was not significantly different from the wild type, and the protein structure around the catalytic dyad was also similar to that of the wild type. On the other hand, E166A (B chain) affected the positions of two neighboring residues, the N-terminal residue S1 (A chain) and H172 (B chain), which altered the shape of the pocket. In fact, the β-strand changed direction at the E166 (A166 in the mutant) and H172, affecting the position of other active site residues such as Q189. In addition, there was a decrease in the frequency of hydrogen bonding in the A chain of the E166A mutant, where the ligand RMSD was not so large. This data also indicates that the mutation E166A affects inhibitor recognition, similar to mutant H163A. Although E166 of A and B chains formed hydrogen bonds between S1 of B and A chains in wild type, respectively, these hydrogen bonds were not observed in the E166A mutant. However, the E166A mutation did not significantly affect the dimeric structure. In addition, the ligand substantially fluctuated thorough the simulations, however 3D structure of the catalytic dyad in E166A mutant was similar to that in wild type. Previous study using SARS-CoV have reported that the E166A mutant form a dimer, and that the hydrogen bond between E166 and S1 is not essential for dimer-structure formation [13]. In addition, E166A mutant is enzymatically active (the enzymatic activity of E166A mutant is about one-third that of the wild type). Our results show that both E166A mutants of SARS-CoV and SARS-CoV-2 have similar structural features.

In the final structure of MD simulation, the ligands in A and B chains in wild type formed four and five hydrogen bonds with the surrounding residues, respectively. In contrast, the ligands in A and B chains in H163A both form three hydrogen bonds with the surrounding residues, and the ligands in A and B chains in Q189A formed three and two hydrogen bonds, respectively. Therefore, the changes of ligand-binding free energy in these mutants are considered to be smaller than that in wild type. In addition, when two heavy atoms are within 4.0 Å, these heavy atoms are defined as “contacting”. In wild type, the pairs of heavy atoms in “contact” between ligand and protein in A and B chains were 69 and 91, respectively. In L141A, N142A, H163A, H164A, and P168A, the number of pairs of heavy atoms in “contact” were reduced by 10 or more compared to that in wild type. In these mutants, there are less hydrophobic contacts between ligand and proteins, and the change of ligand-binding free energies are considered to be smaller than that of wild type.

## 3. Conclusions

In this study, we performed a virtual alanine scan to predict what residues may be important for ligand recognition in SARS-CoV-2 Mpro and do not cause loss of Mpro enzymatic activity when mutated. For the calculations, the complex of Mpro and its inhibitor NCL-00024905 was used. In the complex, NCL-00024905 was bound to the substrate-binding site in Mro, and NCL-00024905 formed hydrogen bonds with F140, H163, E166, and Q189. NCL-00024905 can inhibit the enzymatic reaction of Mpro by preventing the substrate from appropriately binding to the active site. Although mutations to residues besides Ala may give different results, Ala serves as a representative example of an “uncharacteristic residue” and is commonly used to determine the importance of a particular residue in a protein. In SARS-CoV-2 Mpro, mutations H163A and E166A affected ligand recognition but did not change the structure of the catalytic dyad, suggesting that these mutants may weaken the affinity of the inhibitor without a loss in enzymatic activity. In addition, the calculated structure of C145A, whose structure has been obtained experimentally, did not change significantly, despite mutation of the active center residue, consistent with experimental results. Thus, the virtual alanine scan used in this study is expected to be a useful tool for estimating the effects of amino acid mutations on ligand recognition and activity and ultimately in predicting drug resistance.

## 4. Materials and Methods

In this study, the crystal structure of SARS-CoV-2 Mpro (PDB ID: 5RG1) [18] was used. Mpro was assumed to be a homodimer, and MD simulations were performed for the structure in complex with the non-covalent inhibitor NCL-00024905 as the ligand. The ligand was placed in both active sites of the homodimer. The crystal structure was used as the initial structure of the wild type, and the initial mutant structure was the structure after the MD simulation of the wild type with amino acid mutations was introduced. As a model of amino acid mutation, we constructed mutant proteins with one residue in the active site mutated to alanine in silico. By comparison the computational structures of wild-type and mutated protein, the role of pre-mutated amino acid residues in Mpro can be estimated. This method is called a virtual alanine scan. For the virtual alanine scan, all residues within a radius of 4 Å from the ligand were included. The residues that were mutated from the wild-type structure are shown in Figure 1. The assignment of the force field parameters for the ligand and atomic charge calculations was performed using the antechamber module of AmberTools18. The AMBER ff14SB force field [27] was used as the protein force field, and the General AMBER Force Field [28] was used for the ligand. For the explicit solvent model, the cyclic boundary box was spaced with a margin of at least 8 Å from the protein surface, and the TIP3P water molecules filled the box. Sodium ions were placed as a counter ion. Before the MD simulations, 1000-step structural optimizations were performed only for waters and counter ions, and 2500-step minimizations were carried out for the whole system. After the structural optimization, temperature increasing MD simulations were conducted from 0 to 300 K. For the temperature increasing MD simulations, the structure of the complex was fixed, and MD simulations were performed for 20 ps. The equilibration MD simulations were performed for 1000 ns for the wild type and 100 ns for the mutant. The time step of MD simulations was set to 2 fs, and the simulation temperature was 300 K. The snapshots were output every 2 ps. The SHAKE algorithm [29] was employed to constrain bond stretching involving hydrogen atoms. AMBER18 software [30] was used for all classical mechanical calculations.

To analyze the results of MD simulations, we calculated root mean square deviations (RMSDs) for MD trajectories using the initial structure of the equilibrated MD as the reference structure. In addition to the RMSDs of the protein main chain, the RMSDs of the ligand and the catalytic dyad (His41 and Cys145) were determined. The main-chain RMSDs were used to evaluate the convergence of the MD simulations as well as to evaluate changes in the overall structure. The ligand RMSDs were used to evaluate whether the inhibitor could be located in Mpro, and the RMSDs of the catalytic dyad were used to estimate the possibility of loss of enzymatic activity. The ligand RMSDs and catalytic dyad RMSDs were calculated for both homodimeric ligand and catalytic dyad, respectively. In addition to the RMSDs, hydrogen bonding analyses were performed for the final 10 ns portion of the MD trajectory. Hydrogen bonds were detected by focusing on the hydrogen bonds between the protein and the ligand, using a bond distance of 3.5 Å and a bond angle of 120° as a threshold. The root mean square fluctuations (RMSFs) were calculated using the final 10 ns of the MD trajectory to quantify the fluctuations in protein structure. For RMSF calculations, the average structure of the final 10 ns trajectory was used as the reference structure, and the RMSFs of the α-carbon of each amino acid residue were calculated. The cpptraj module of AmberTools18 was used for the analyses.

## Figures and Tables

**Table 1:** Artificial mutants used for virtual alanine scan.

F140A	L141A	N142A	S144A	C145A
H163A	H164A	M165A	E166A	L167A
P168A	H172A	D187A	R188A	Q189A

**Table 2:** Averaged RMSDs/Å for the last 10 ns MD trajectories of artificial mutants.

	Main Chain	Ligand A	Ligand B	Catalytic Dyad A	Catalytic Dyad B
F140A	1.445	1.002	1.264	1.258	1.269
L141A	1.413	1.350	1.054	0.685	1.197
N142A	1.375	**1.970**	0.895	1.083	1.024
S144A	**1.537**	**1.716**	1.332	1.108	1.203
C145A	1.387	1.190	1.031	0.848	1.155
H163A	1.402	1.308	2.315	1.188	0.930
H164A	1.176	1.165	0.997	0.741	0.949
M165A	**1.753**	1.421	**1.617**	1.094	1.334
E166A	1.282	0.956	2.293	1.179	0.793
L167A	1.246	1.181	1.270	0.747	0.888
P168A	1.283	1.176	1.009	0.722	1.132
H172A	1.199	0.979	0.923	**1.780**	1.080
D187A	1.288	1.367	**1.637**	**1.715**	2.121
R188A	1.202	1.173	**1.584**	0.707	1.023
Q189A	1.460	1.200	1.113	1.204	1.156

Solid underline: >2.0 Å; bold: >1.5 Å.

## Data Availability

Data is contained within the Article.

## Supplementary Materials

The following are available online at https://www.mdpi.com/article/10.3390/ijms22189837/s1.
