# Supplementary material for: Virtual Alanine Scan of the Main Protease Active Site in Severe Acute Respiratory Syndrome Coronavirus 2
Source: Int J Mol Sci. 2021 Sep 11;22(18):9837. doi: 10.3390/ijms22189837 (PMC8466562; doi:10.3390/ijms22189837)
Supplement: Supplementary file 1 [file ijms-22-09837-s001.zip › ijms-1360328-supplementary.pdf]

Supplementary Materials: Virtual alanine scan of severe acute respiratory syndrome coronavirus 2 main protease active site

Tomoki Nakayoshi [1,2], Koichi Kato [2,3], Eiji Kurimoto [2] and Akifumi Oda [2,4,*]

[1] Graduate School of Information Sciences, Hiroshima City University, 3-4-1 Ozukahigashi, Asaminami-ku, Hiroshima, Hiroshima 731-3194, Japan; nakayoshi@hiroshima-cu.ac.jp (T.N.)
[2] Faculty of Pharmacy, Meijo University, 150 Yagotoyama, Tempaku-ku, Nagoya, Aichi 468-8503, Japan; k-kato@kinjo-u.ac.jp (K.K.); kurimoto@meijo-u.ac.jp (K.K.)
[3] Faculty of Pharmaceutical Sciences, Shonan University of Medical Sciences, 16-48 Kamishinano, Totsuka-ku, Yokohama, Kanagawa 244-0806, Japan
[4] Institute for Protein Research, Osaka University, 3-2 Yamadaoka, Suita, Osaka 565-0871, Japan
* Correspondence: oda@meijo-u.ac.jp; Tel.: +81-52-832-1151

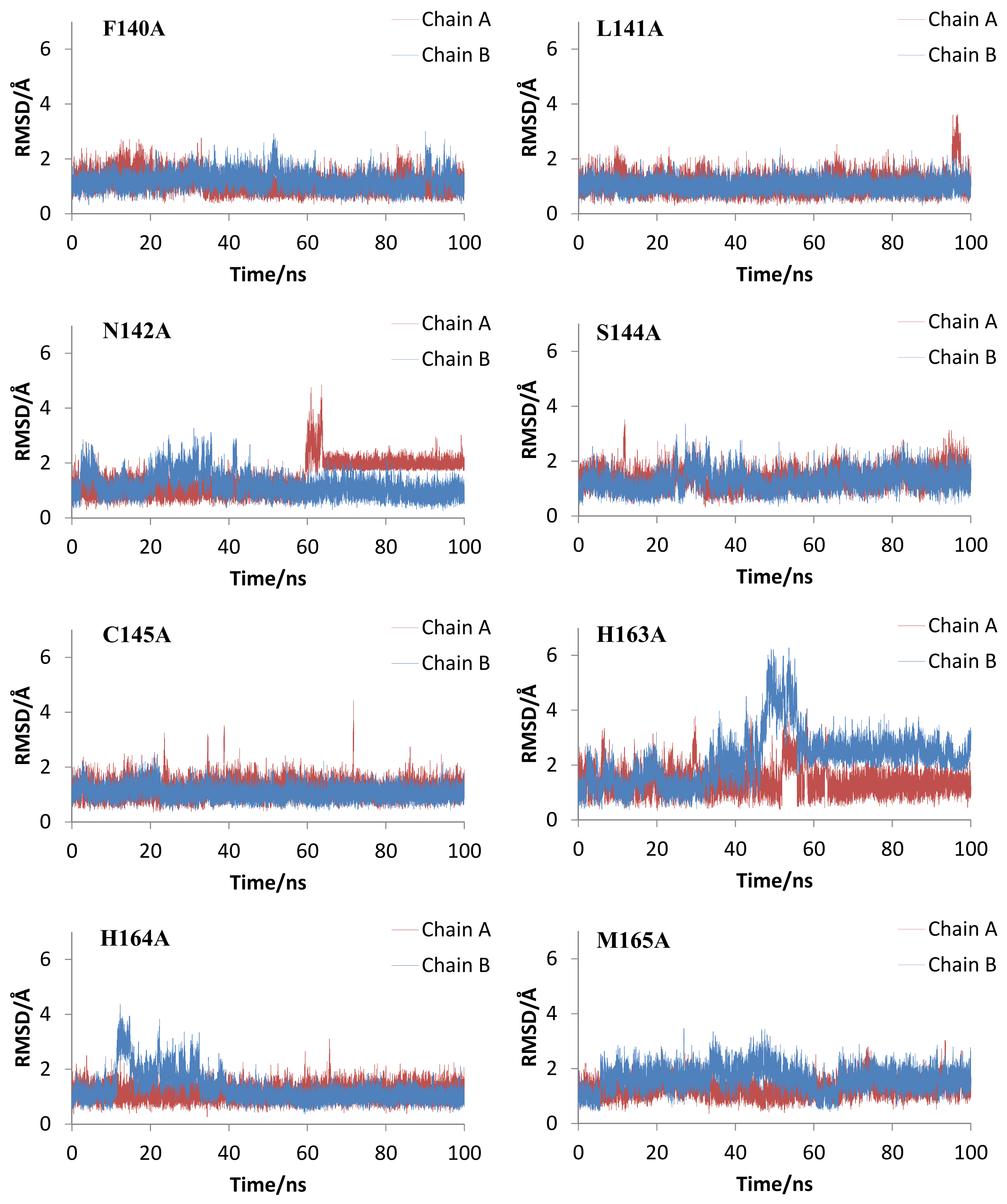

Figure S1. Ligand RMSDs for virtual alanine scanned mutants.

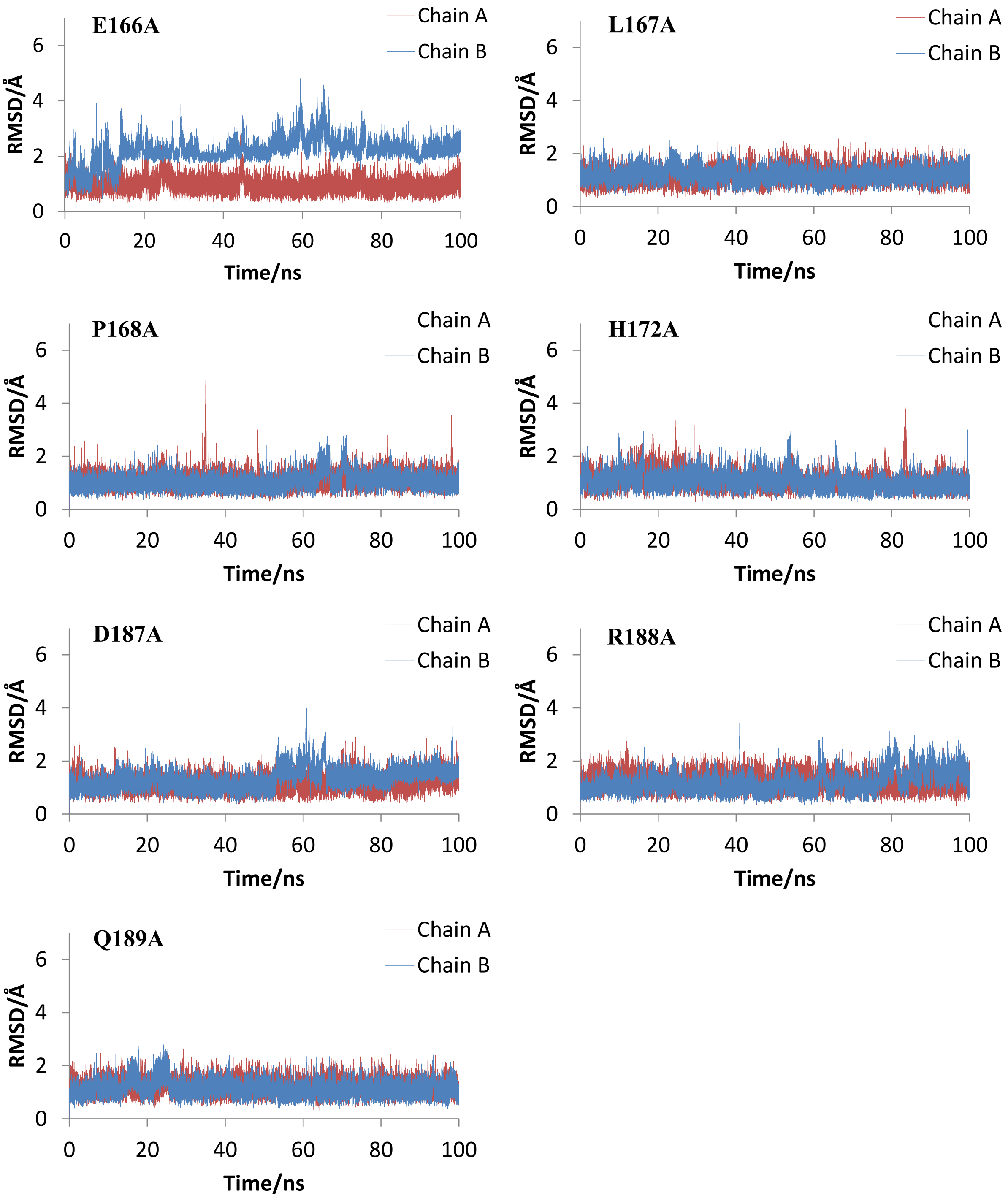

Figure S1. Continued.

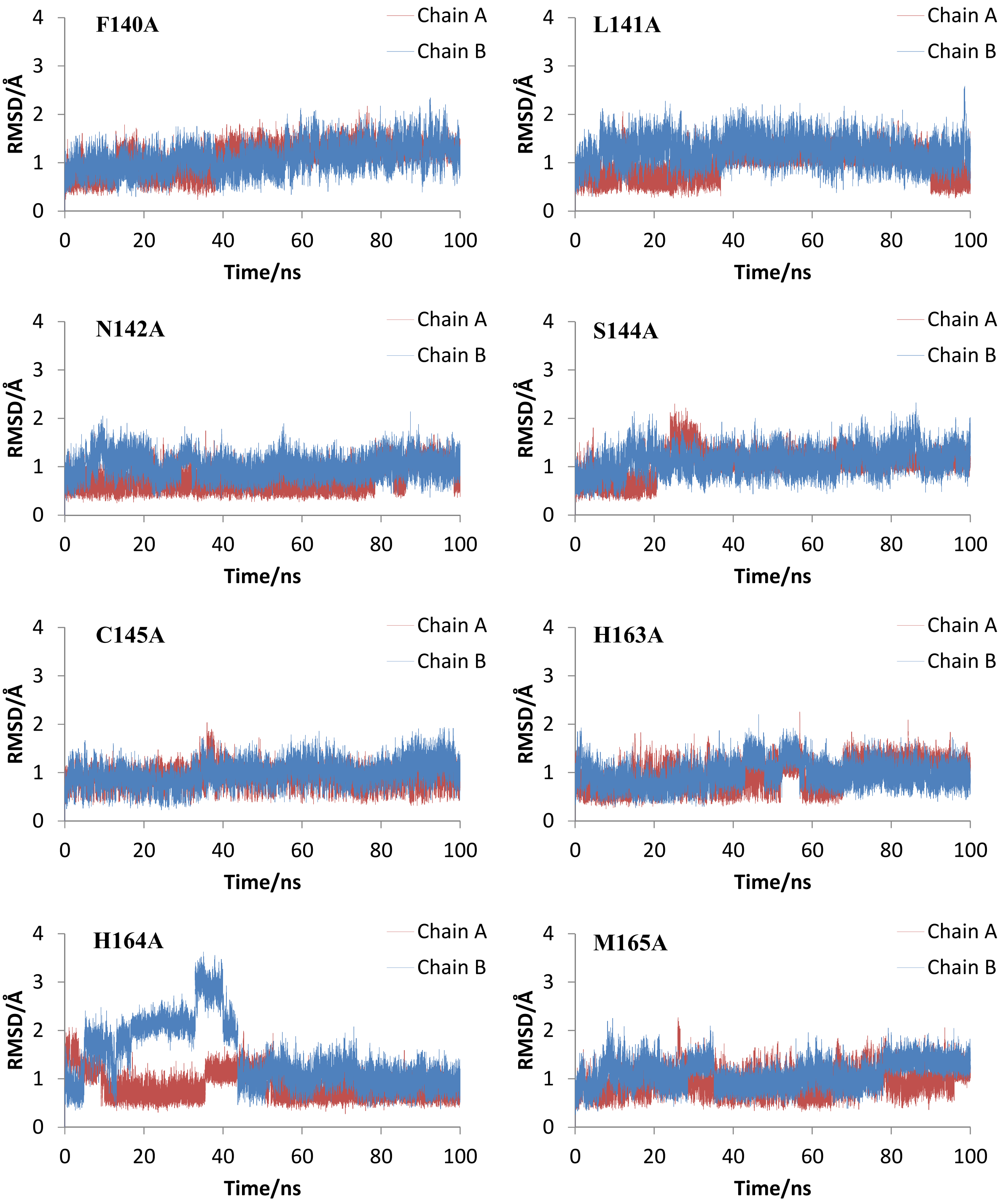

Figure S2. Catalytic dyad RMSDs for virtual alanine scanned mutants.

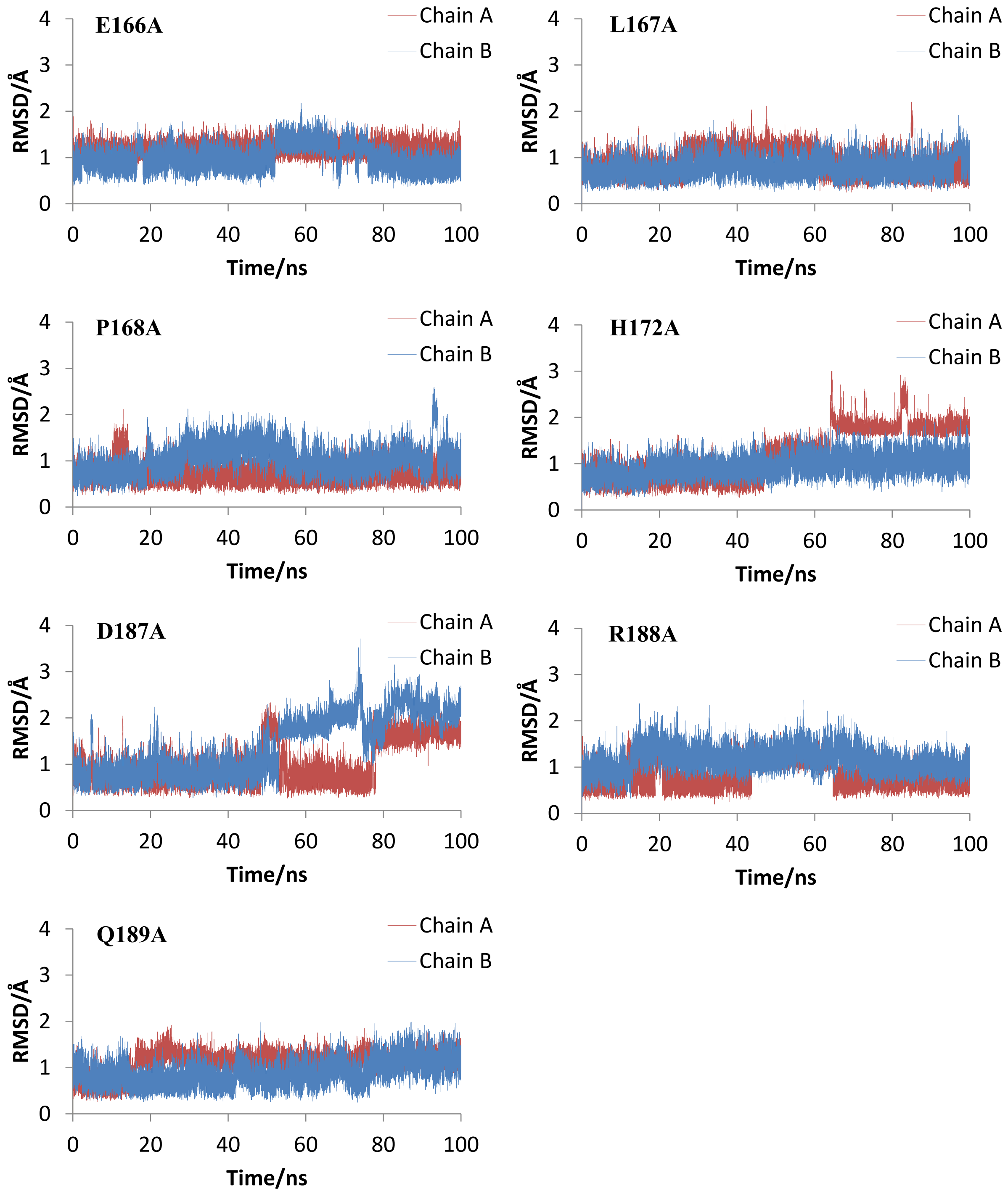

Figure S2. Continued.
